# Hierarchical analysis of ontogenetic time to describe heterochrony and taxonomy of developmental stages

**DOI:** 10.1038/s41598-020-76270-4

**Published:** 2020-11-12

**Authors:** Guillaume Lecointre, Nalani K. Schnell, Fabrice Teletchea

**Affiliations:** 1 Institut de Systématique, Évolution, Biodiversité (ISYEB), UMR 7205 Muséum national d’Histoire naturelle, CNRS, SU, EPHE, UA, Sorbonne Universités, CP24, Muséum national d’Histoire naturelle, 57 rue Cuvier, 75005 Paris, France; 2 Institut Systématique, Évolution, Biodiversité (ISYEB), UMR 7205 Muséum national d’Histoire naturelle, CNRS, SU, EPHE, UA, Sorbonne Universités, Station Marine de Concarneau, Place de la Croix, 29900 Concarneau, France; 3 Université de Lorraine, Unité de Recherche Animal and Fonctionnalités des Produits Animaux, Institut national de recherche pour l’agriculture, l’alimentation et l’environnement, 54505 Vandœuvre-lès-Nancy, France

**Keywords:** Developmental biology, Evolution, Zoology

## Abstract

Even though an accurate description of early life stages is available for some teleostean species in form of embryonic and post-embryonic developmental tables, there is poor overlap between species-specific staging vocabularies beyond the taxonomic family level. What is called “embryonic period”, “larval period”, “metamorphosis”, or “juvenile” is anatomically different across teleostean families. This problem, already pointed out 50 years ago, challenges the consistency of developmental biology, embryology, systematics, and hampers an efficient aquaculture diversification. We propose a general solution by producing a proof-of-concept hierarchical analysis of ontogenetic time using a set of four freshwater species displaying strongly divergent reproductive traits. With a parsimony analysis of a matrix where “operational taxonomic units” are species at a given ontogenetic time segment and characters are organs or structures which are coded present or absent at this time, we show that the hierarchies obtained have both very high consistency and retention index, indicating that the ontogenetic time is correctly grasped through a hierarchical graph. This allows to formally detect developmental heterochronies and might provide a baseline to name early life stages for any set of species. The present method performs a phylogenetic segmentation of ontogenetic time, which can be correctly seen as depicting ontophylogenesis.

## Introduction

Even though an accurate description of early life stages is available for a few teleostean species in form of embryonic or post-embryonic developmental tables (e.g. for clownfish *Amphiprion ocellaris*^1^, and for zebrafish *Danio rerio*^2^), there is poor overlap between species-specific staging vocabulary beyond the taxonomic level of family^3^. For instance, Peñáz^4^ showed that the development of anatomical characters during the “larval period” differ in Coregonidae, Thymallidae, Osmeridae and Salmonidae, all families of the same order. The difficulty of a common staging vocabulary is therewith considerable among teleosts, which is by far the most speciose taxon within vertebrates (more than 32,000 species^5^) along with a remarkable diversity of reproductive traits^6,7^. For instance, the onset and the end of the “larval period”^4,8^ is still a matter of strong discrepancy, as convincingly exposed by Urho^3^. Criteria by which a teleostean “larva” is defined have never been consensual^8–17^. And beyond teleosts, Haug^18^ recorded seven different criteria used in zoology to identify what is named “a larva” throughout the animal diversity. What is a larva in non-amniotic vertebrates? The question remains fundamental in zoology, embryology, evo-devo, ecology and aquaculture. On the long term, the scientific and economic consequences of these conceptual limits may be potentially negative, tough not precisely assessed.

Finding a common vocabulary to describe the early development in vertebrates is difficult because there is no formalized method to compare and consistently name early life history stages. However, having the ability to apply a common language and concept is important in order to avoid that the same terminology might be applied to stages of two species with different anatomical development, leading to confusion regarding this terminology of stages as pointed out by Urho^3^. Without a common terminology based on common concepts, dialogs between e.g., aquaculturists rearing different species are severely limited^19^, especially in transferring rearing practices from one species to another beyond the taxonomic limit of families^20,21^. Such a situation hampers the consistency of biology, comparative anatomy and embryology: it is not only a matter of naming the same way the “same” phenotypic character^22,23^ or the “same” developmental event^24^, it is a matter of consistently naming stages of organismal development as well. At last, Ecology could be impacted when we need to grasp the role and dynamics of “fish larvae” as a whole^25,26^ with the potential risk to mix entities at heterogeneous developmental stages across species.

The existing terminologies are limited in their usefulness and capacity to compare early life history stages in teleostean vertebrates. One terminology example is the ontogenetic index^27^, another example is based on the degree of flexion of the terminal section of the notochord during caudal fin development, separating pre-flexion, flexion and post-flexion stages^8,28,29^, especially useful for identification of marine teleostean larvae.

However, such staging terminologies have the disadvantage of not taking into account evolutionary processes, which is important because terminology cannot be solely descriptive, it has to be explanatory^30,31^. For example, Werneburg’s^24^ work attempted to standardize developmental events across the diversity of vertebrates, but this was not performed under the principles of phylogenetic systematics^20^, which is however crucial because ontogeny is linked to phylogeny. Defining developmental stages in the frame of phylogenetic systematics of Hennig^32,33^ requires to design and name stages under the form of nested sets. However, this has never been tackled so far^34^. For instance, there has been much effort made to understand and analyse developmental characters and developmental sequences within a phylogenetic framework^35–37^ and subsequent studies^38–40^ focused on the detection of heterochronies and the phylogenetic usefulness of developmental characters. However, none of them dealt with staging terminologies. Various authors tried to link gene ontologies to phenotypic ontologies within an evolutionary framework^22,23^. But the issue of circumscribing and naming developmental stages of the organism was not fully conducted. The present work proposes a method to define developmental stages from *as many anatomical traits as possible* for *as many species as possible*—e.g. in the frame of modern multi-species comparative methods—in the frame of phylogenetic systematics.

### Why is there no general method for naming developmental stages?

There are neither universal criteria nor a method for defining any developmental stage *in general*. Ontogenetic comparison across teleostean species were rarely investigated and suffered from limitations^4,27^ like:

- Most authors refer to ecology and adaptation to make sense of huge differences in the tempo of rise of developmental events and traits across teleostean species. The ontogenetic index^27^ was a good start for formalization of comparison, however it has been developed outside an evolutionary framework and phylogenetic systematics, and was barely used in the past decades (Fuiman, personal communication). Standard comparative methods in the realm of phylogenetic systematics were not used.
- Inter-individual variability^27^;
- Dependence on temperature^41,42^;
- Character rise or developmental events occur at different organismal sizes across species^3^;
- Heterochronic development^43^.

### Heterochronic development in teleostean fishes

Fuiman^44^ detected that some morphological features appeared earlier during the development of flatfishes than in pelagic species, especially for the sensory, locomotor and digestive systems. Fuiman^45^ tried to construct a phylogeny of the teleostean family Catostomidae based on larval traits (including relative timing of developmental events) and found that (1) the phylogeny of species based on larval characters failed to recover some of the groups found with adult characters and (2) several characters showed a phylogenetic acceleration of development—showing heterochrony. Shardo^43^ showed that the American shad *Alosa sapidissima* displayed delayed hatching, neuromasts, and opercular development relative to other clupeoids. Shardo also made clear that the concept of heterochrony cannot be avoided when one tries to clearly establish developmental stages not only useful for a single species but for several. *The study of heterochrony is therefore part of the problem of defining developmental stages shared across species*^34^. Further, heterochrony is also a key process that generates a large variety of forms and life history traits and as such, require an objective method to be properly assessed. For example, heterochrony explains the diversity of jaw morphology within the family of needlefishes (Belonidae) from halfbeak to needlenose^46^. A comparative method designed to name developmental stages has to face the phenomenon of heterochrony. The method we propose within this paper is unique as it is able to detect the phenomenon of heterochrony.

### Better accuracy and wider validity of developmental stages would provide a better approach to metamorphosis

One of the landmarks for defining the end of the larval period is metamorphosis (for instance in Fuiman’s^27^ ontogenetic index). However, there is still no clear and general concept of metamorphosis suitable for all non-amniote vertebrates. We already knew that what is called “hatching”—another landmark event—has no meaning with regard to the degree of development of organs in teleosteans^47–49^. In other words, depending on the species, the hatching larva can either be already well developed (with an open mouth to feed, certain fins developed) or rather less developed (mouth closed, large yolk sac, no fins developed). Campinho^50^ stressed that “*teleost metamorphosis is still an understudied developmental event*.” Thyroid hormone signaling is nevertheless a universal feature. TH signaling triggers metamorphosis in sea urchins, urochrodates (*Ciona*), cephalochordates, teleosts^17^ that then undergo strong ecological transitions; and it is relevant to note that there is a TH rise at hatching of avian eggs and at birth of mammals^17^. Is metamorphosis a universal post-embryonic transition? Is this transition universally controlled by TH signaling? We need a unified conceptual framework to rigorously address this. The larval stage needs to be more precisely defined by the present comparative multi-species approach before being compared to the timing of TH signaling.

### How to measure heterochrony and name stages in a multi-species framework?

The goals of this paper are (1) to detect heterochronies; (2) to offer the possibility to name in the same way developmental stages of any set of species by producing a hierarchization of ontogenetic time. The starting point is a matrix where columns are characters (presence of organs and traits) and lines a given species at a given time; i.e. a matrix of across-species comparisons of characters available at different degree-days of their development (temperature*time, with temperature in degree Celsius (°C) and time in days)^21^. This is followed by a standard parsimony analysis of that matrix. No reference phylogeny is necessary for our method; the only requirement is data on presence or absence of a given organ in a given taxon at a given time. Names given to ontogenetic stages will get a wider domain of validity and heterochronies will be objectively detected.

## Results

**Figure 1:**
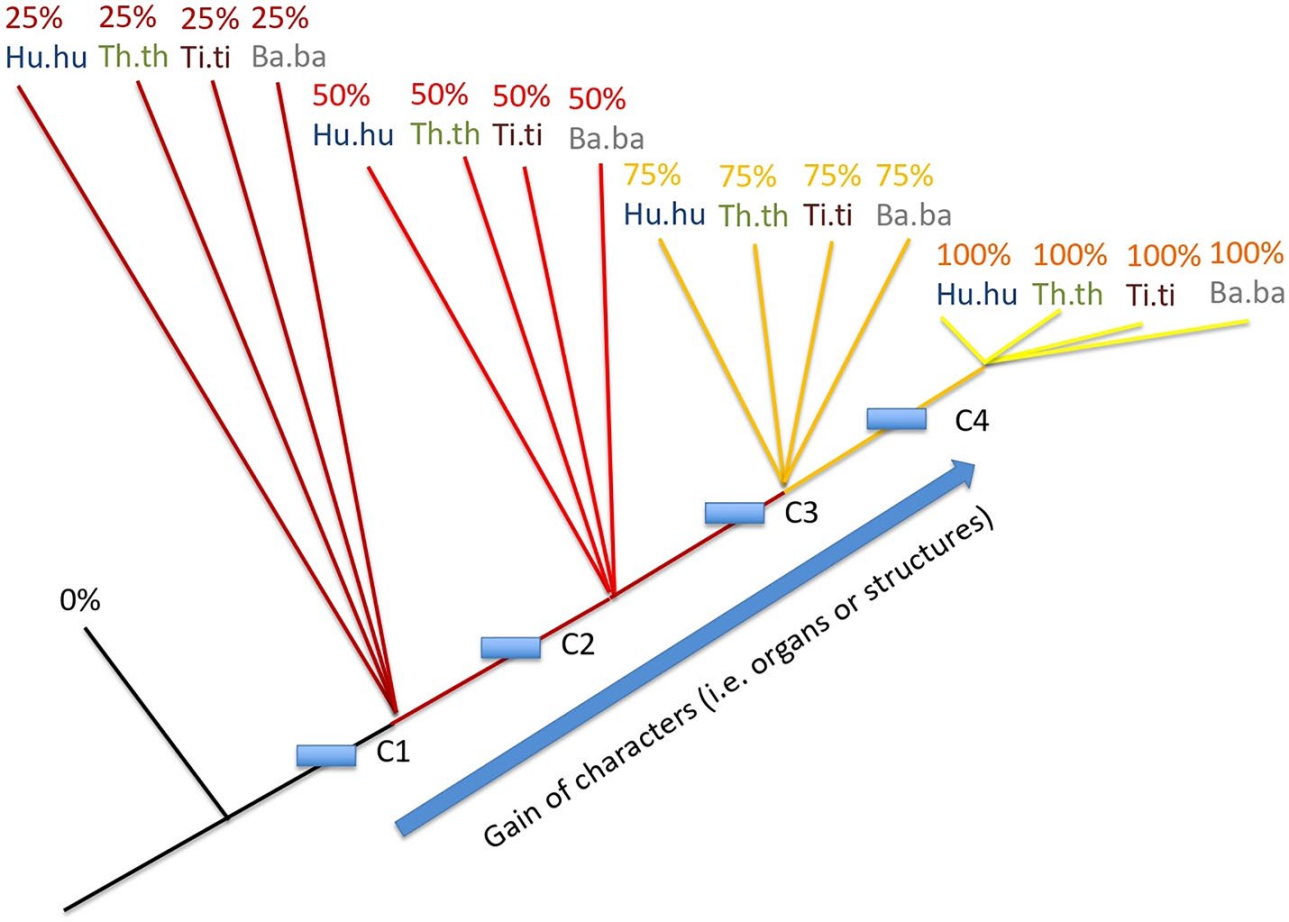
Hierarchical structure expected for our OTUs through time frame 25–50–75% if the relative timing of the onset of characters for the four species were the same (“synchronic” development).

**Figure 2:**
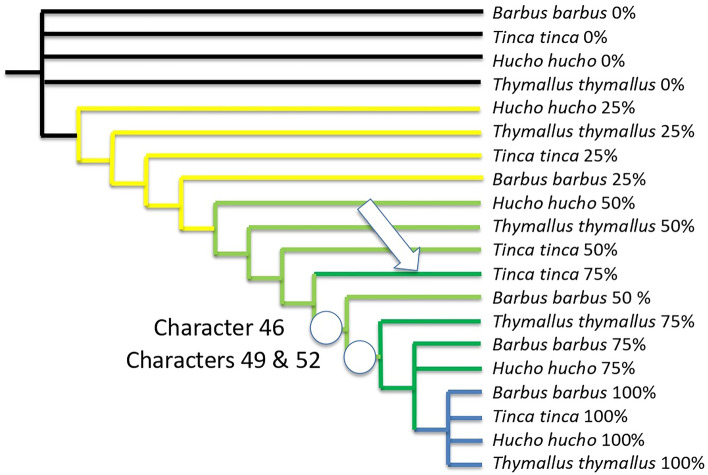
Strict consensus of two equi-parsimonious trees with the length of 62 steps, consistency index of 0.85 and retention index 0.96, obtained for the four species under the time frame 25–50–75%. Note that *Hucho* is late compared to *Thymallus*, *Thymallus* is late compared to *Tinca,* and *Tinca* is late compared to *Barbus*. The arrow shows heterochrony (see text); onsets of characters shown with circles are those detecting it.

Under a “synchronic development” using the time frame 25–50–75% among the four species, we should obtain the theoretical tree shown in Fig. 1. The parsimony analysis of the 53 characters (Tables 1 and 2) considered through this same time frame for the four species results in two equi-parsimonious trees with the length of 62 steps (strict consensus shown in Fig. 2), with a consistency index of 0.85 and retention index of 0.96. Such high consistency values mean that the developmental time is a hierarchical time, in other words (1) the rise of organs is overall cumulative and (2) their timing is much similar between the four species.

**Table 1:** Data matrix.

Barbus_barbus_0%	00000000000000000000000000000000000000000000000000000
Tinca_tinca_0%	00000000000000000000000000000000000000000000000000000
Hucho_hucho_0%	00000000000000000000000000000000000000000000000000000
Thymallus_thymallus_0%	00000000000000000000000000000000000000000000000000000
Barbus_barbus_5%	1111111111111110000000?000000000000000000000000000000
Barbus_barbus_10%	1111111111111111111111?000000000000000000000000000000
Barbus_barbus_20%	1111111111111111111111?111111111110000000000000000000
Barbus_barbus_25%	1111111111111111111111?111111111111110000000000000000
Barbus_barbus_50%	1111111111111111111111?111111111111111111111111000000
Barbus_barbus_75%	1111111111111111111111?111111111111111111111111111100
Barbus_barbus_100%	1111111111111111111111?111111111111111111111111111111
Tinca_tinca_5%	11111111111111111101100000000000000000000000000000000
Tinca_tinca_10%	11111111111111111101110101111000000000000000000000000
Tinca_tinca_20%	11111111111111111111111111111111010100000000000000000
Tinca_tinca_25%	11111111111111111111111111111111010100010000000000000
Tinca_tinca_50%	11111111111111111111111111111111111111011010101100000
Tinca_tinca_75%	11111111111111111111111111111111111111111111101100000
Tinca_tinca_100%	11111111111111111111111111111111111111111111111111111
Hucho_hucho_5%	11111111100000000000000000000000001000000000000000000
Hucho_hucho_10%	11111111111100000000000000000000001000000000000000000
Hucho_hucho_20%	11111111111111111101100010000000001000000000000000000
Hucho_hucho_25%	11111111111111111101110010111000001000000000000000000
Hucho_hucho_50%	11111111111111111111111111111111111111110100010010000
Hucho_hucho_75%	11111111111111111111111111111111111111111111111111110
Hucho_hucho_100%	11111111111111111111111111111111111111111111111111111
Thymallus_thymallus_5%	1111111111000000000000000000?000?000?000?000000000000
Thymallus_thymallus_10%	1111111111111111000000000001?000?000?000?000000000000
Thymallus_thymallus_20%	1111111111111111111111001111?011?100?000?000000000000
Thymallus_thymallus_25%	1111111111111111111111111111?011?100?100?000000000000
Thymallus_thymallus_50	1111111111111111111111111111?111?111?111?110000000000
Thymallus_thymallus_75%	1111111111111111111111111111?111?111?111?111111110010
Thymallus_thymallus_100%	1111111111111111111111111111?111?111?111?111111111111

Columns: Characters (i.e. organs, traits) as listed in Table 2. “0” means absence, “1” means presence, “?” means unavailable data.

**Table 2:** List of characters, obtained from Peñáz^69–71^, Peñáz and Prihoda^72^, and Krupka^73^.

Number	Characters
1	Fecundation
2	Perivitelline space
3	Bipolar differentiation
4	Blastodisc
5	Cleavage
6	Two blastomeres
7	Four blastomeres
8	Morula
9	Blastula
10	Gastrula
11	Neurulation
12	Somites (around 10)
13	Rudimentary heart
14	Rudimentary eyes
15	Brain begins
16	Tail bud
17	Eye lenses
18	Vibrations of muscle
19	Full number of somites
20	Pulsating heart
21	Development of embryonic finfold
22	Segmentation of the caudal part completed
23	Hatching glands
24	First hatching
25	Rudiment of the pectoral fins
26	Otoliths appear
27	Development of vena caudalis inferior
28	Separation of front of head from the yolk sac
29	Development of the ducti Cuvieri on yolk sac
30	Last hatching
31	Complete separation of head from yolk sac
32	Pigment of the eye
33	Development of semicircular canals
34	First pigmentation on body
35	Lower jaw starts moving
36	Development of gill lamellae
37	First movements of pectoral fins
38	Mouth opening
39	Development of lepidotrichia in the caudal fin
40	Branchial respiration started
41	Gas bladder filled with gas
42	Finfold begins to differentiate into zones of individual unpaired fins
43	Start exogenous feeding
44	Respiration function fully taken by gills
45	Yolk has completely disappeared
46	Development of lepidotrichia in dorsal fin
47	Change to exclusively exogenous feeding
48	Development of lepidotrichia in pectoral fins
49	Development of lepidotrichia in anal fin
50	Complete number of rays in anal fin
51	Complete number of rays in dorsal fin
52	Lepidotrichia developed in the pelvic fin
53	Full number of rays in all fins

Note that at 25% and 50% of their developmental time, *Hucho hucho* was late compared to *Thymallus thymallus*; grayling is late compared to *Tinca tinca*; tench is late compared to *Barbus barbus*. It is interesting that this trend is modified at 75% of the developing time, where *Tinca tinca* is late (white arrow in Fig. 2): at that time segment, it does not yet exhibit characters 46, 49 and 52 in contrast to all other species (46 is the onset of lepidotrichia in dorsal fins, 49 is the onset of lepidotrichia in anal fin, 52 is the onset of lepidotrichia in pelvic fins).

**Figure 3:**
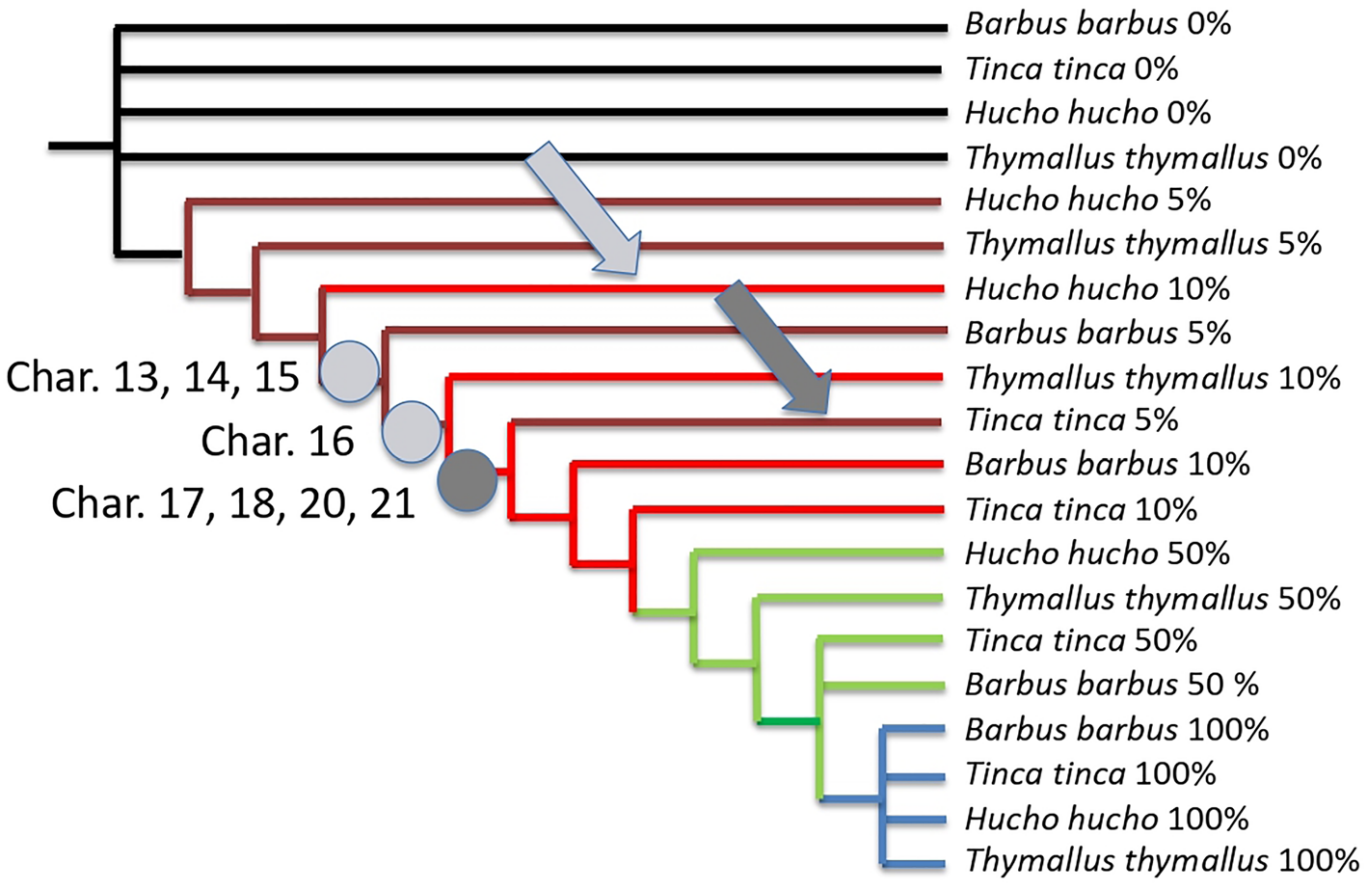
Most parsimonious tree with the length of 62 steps obtained for the four species under the time frame 5–10–50%; consistency index is 0.85 and retention index 0.97. Arrows point out heterochronies: note that *Hucho hucho* is late at 10% of its development (light grey arrow) compared to all other species, and *Tinca tinca* accelerates at the start of its development compared to others (dark grey arrow); onsets of characters shown with circles are those detecting them (see text).

With the time frame 5–10–50%, the parsimony analysis of the 53 characters (Tables 1 and 2) results in a single parsimonious tree with the length of 62 steps (Fig. 3), with a consistency index of 0.85 and retention index of 0.97. Once again, *Hucho hucho* is late compared to all other species. At 10% of its development, *Hucho hucho* is late (light grey arrow Fig. 3) compared to all other species because it does not exhibit yet the characters 13 (rudimentary heart), 14 (rudimentary eyes), 15 (beginning brain) and 16 (tail bud) while others have them at 10%. Note that *Tinca tinca* accelerates at the start of its development compared to others (deep grey arrow Fig. 3), because at 5% it already has characters 17 (eye lenses), 18 (vibration of muscles), 20 (pulsatile heart) and 21 (embryonic finfolds beginning). If we cumulate results, we could say that *Tinca tinca* accelerates at the beginning (5%) and slows down at the end (75%, previous tree).

**Figure 4:**
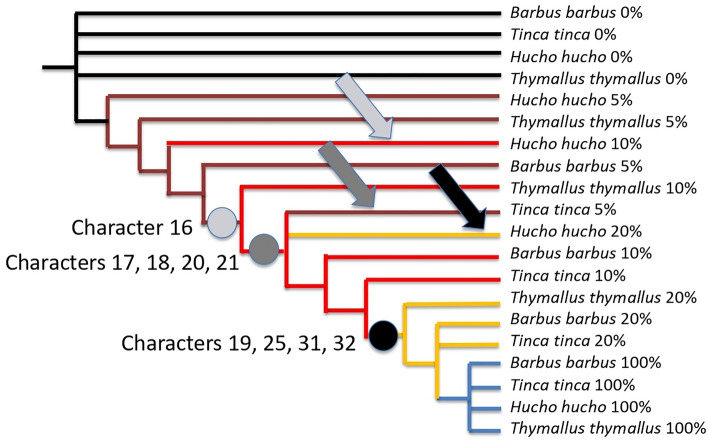
Strict consensus of four trees with the length of 61 steps, consistency index of 0.86 and retention index of 0.96, obtained for the four species under the time frame 5–10–20%. Arrows point out heterochronies: note that *Hucho hucho* is still late at 20% of its development compared to other species (black arrow), because it does not have yet characters 19, 25, 31, 32 (see text).

If we focus even closer on the earliest stages, we confirm that *Hucho hucho* is late compared to other species. With the time frame 5–10–20%, the parsimony analysis of the 53 characters (Tables 1 and 2) results in four parsimonious trees with the length of 61 steps (strict consensus shown Fig. 4), with a consistency index of 0.86 and retention index of 0.96. That tree recovers what we have previously shown : *Hucho hucho* is late at 10%, *Tinca tinca* is in advance at 5%, and here we show that *Hucho hucho* is still late at 20% (black arrow Fig. 4) because it does not exhibit yet characters 19, 25, 31, 32, 34 that others do have at 20% (respectively full number of somites, rudiments of pectoral fins, complete separation of head and yolk sac, pigment of the eye, first pigmentation of the body).

**Figure 5:**
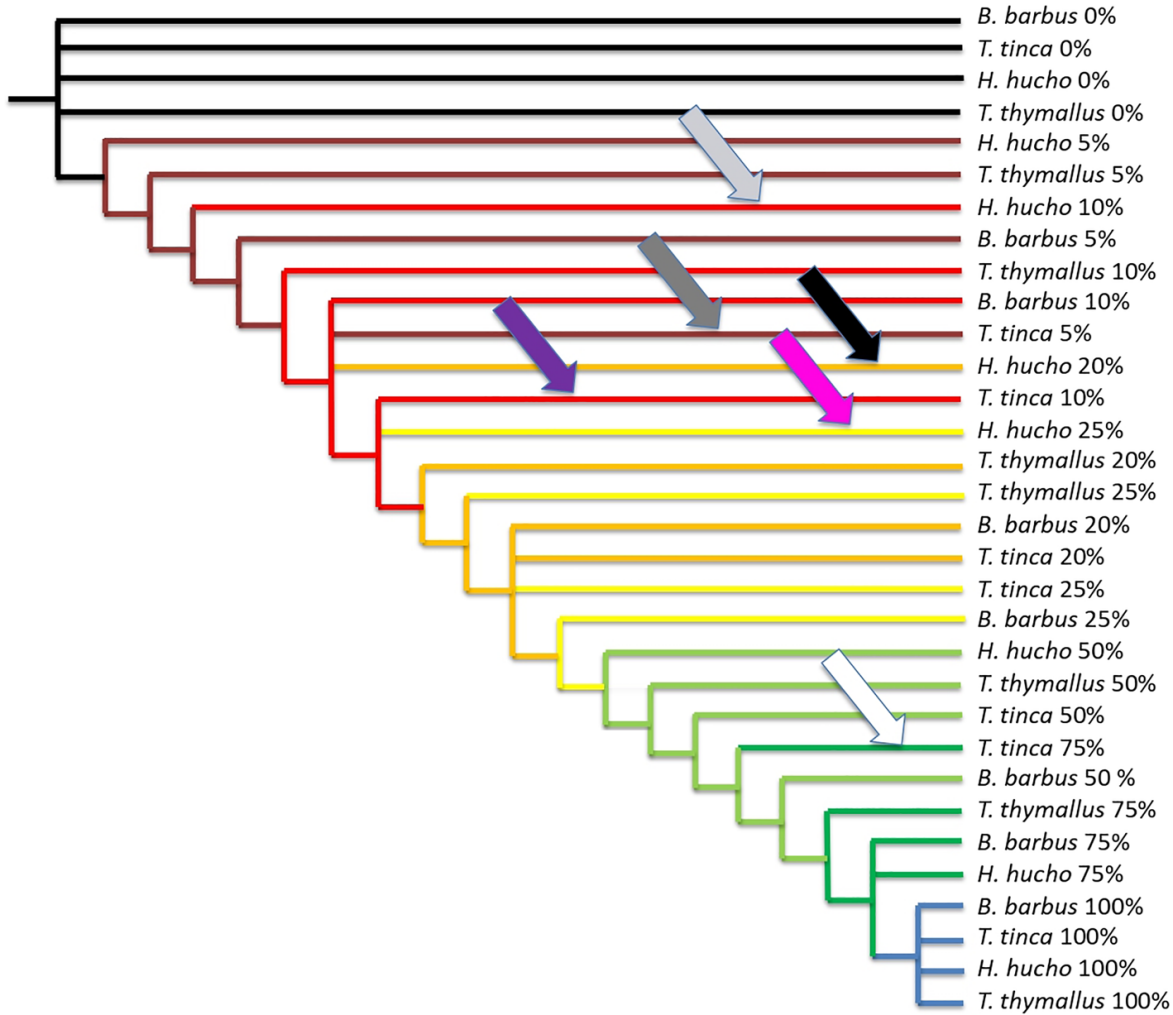
Strict consensus of 50 trees with the length of 71 steps, consistency index of 0.74 and retention index of 0.96, obtained for the four species under the time frame 5–10–20–25–50–75%. Arrows point out heterochronies: all the heterochronies mentioned above are confirmed showing the robustness of the findings when all data are put together. Moreover, the tree highlights the fact that *Tinca tinca* is not only in advance at 5% (deep green arrow) but it is still in advance at 10% (purple arrow); and *Hucho hucho* is not only late at 10% (light grey arrow) and at 20% (black arrow) but it is also late at 25% (pink arrow).

Finally, we wanted to test the robustness of our findings by performing a single parsimony analysis of the 53 characters through the narrowed time frame 5–10–20–25–50–75%, which provides a matrix with 32 OTUs. Parsimony analysis of this matrix generates 50 equi-parsimonious trees with the length of 71 steps with a consistency index of 0.74 and retention index of 0.96. The corresponding strict consensus is shown in Fig. 5. All the results mentioned above are confirmed (white, light grey, deep gray and black arrows: Fig. 5), which shows that the previous results are still robust even when all data are gathered in a single analysis. All the data together allow to display the fact that *Tinca tinca* is not only in advance at 5% (deep green arrow) but it is still in advance at 10% (purple arrow); and *Hucho hucho* is not only late at 10% (light grey arrow) and at 20% (black arrow) but it is also late at 25% (pink arrow).

## Discussion

Here we develop a method to describe heterochronies in a multi-species context. We detected that, overall, *Hucho hucho* is late compared to other species in the earliest developmental periods. This means that this delay should be compensated later, as all species reach 100%. Our method allows to play with time frames, and this hypothesis could be tested by using a time frame of e.g., 50–70–85%. In contrast, *Tinca tinca* accelerates at the beginning but seems to slow down later in development. Such parsimony analyses could be conducted using both more species and time frames with as many time slices as needed. This method could also be used to study intraspecific heterochronies^51^ particularly in link with climate change as it is well known that temperature significantly modify developmental rates^42^ and perhaps developmental patterns among populations.

### Naming stages

**Figure 6:**
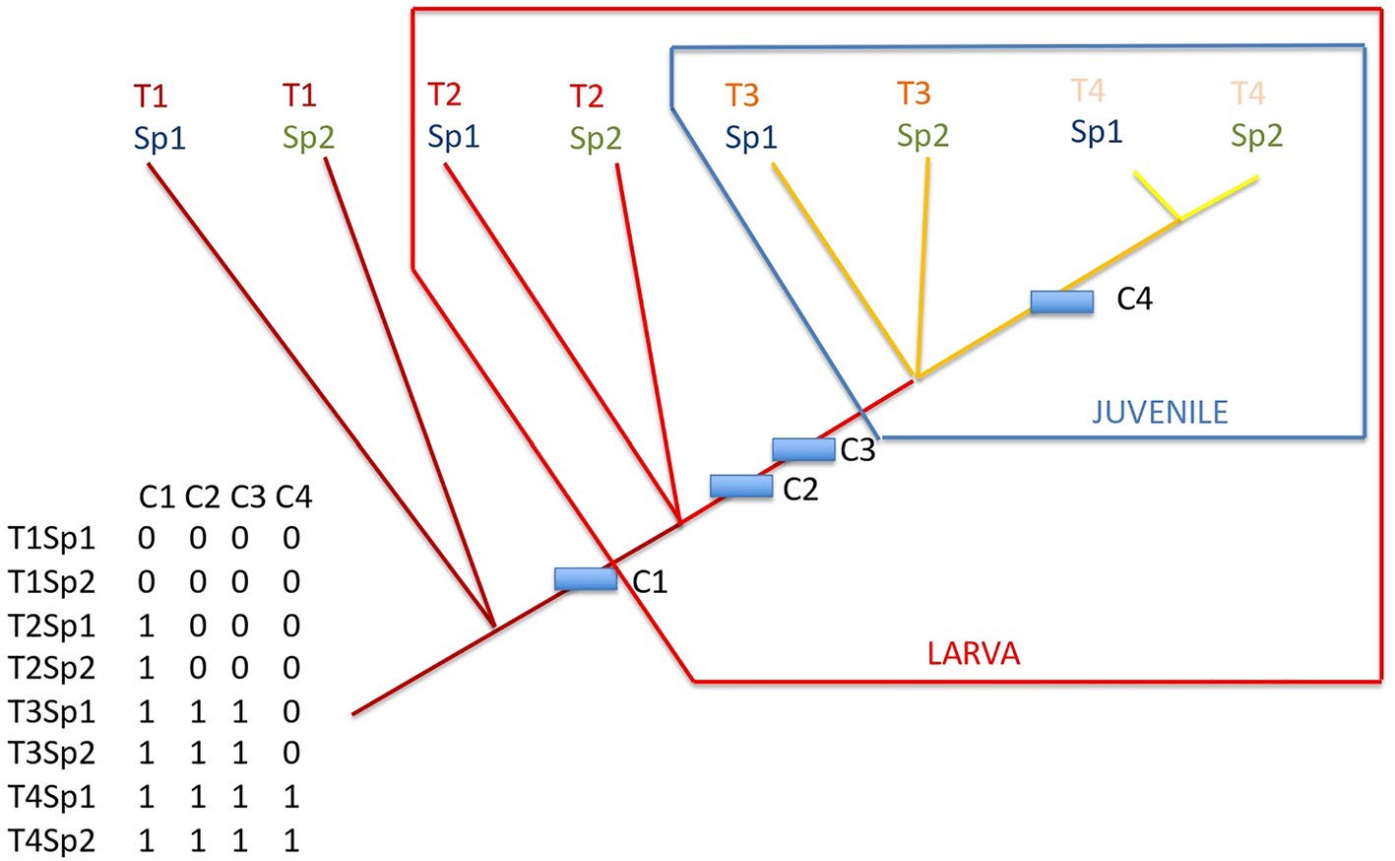
Same theoretical tree as Fig. 7, suggesting nested sets of developmental stages (see text).

Another outcome is a staging terminology. As previously stated, names of developmental stages have been given through separated taxonomic isolates, often inductively restricted to families (Salmonidae, Cyprinidae, etc.). Here we provide the possibility to name stages with a wider validity, because the analytical process on which the stages are based compares species of different families and, possibly, different orders (here we treat Salmoniformes and Cypriniformes, but one could add Characiformes, Percoidei, etc.). Defining the terms “larva”, “juvenile”, or “metamorphosis” is beyond the scope of the present proof-of-concept paper. In Fig. 6, we propose a way to name stages. A given node could be chosen to propose an arbitrary name. It is important to emphasize that (1) those names are valid for a wide taxonomic scope (actually the one covered by the species sampling) because based on an explicit and formal comparative method and (2) as the developmental time is a hierarchical time, stages are defined as nested sets. Indeed, to be self-consistent, a concept (a developmental stage) must contain all the entities that have the attributes by which it has been defined. For instance, it would not make sense to justify the set of mammals by the sharing of hairs and a single jaw-bone, while leaving some entities having hairs and a single jaw-bone outside mammals. Consistency of our language depends on completeness of our concepts. In traditional ways to segment time, the larva was defined by having the organ X, then the juvenile was defined by having later another organ Y (non-homologous, i.e. somewhere else in the organism). Doing so, the animal, when considered as a juvenile, was no longer considered as a larva. It is inconsistent, because the organ X by which the larva was defined is most often still there in the juvenile, and even in the adult (such as eyes, fin rays…). Developmental stages should not be segmented in an exclusive manner as series of successive sets, but segmented in an inclusive manner, i.e. should be designed as nested sets. It is a gain in conceptual consistency, and not a loss in usefulness. Indeed, an individual fish of the species 2 at the time 3 (Fig. 6), would be considered a juvenile. The fact that it is also still a larva is just a cumulative, ancestral property that does not need to be mentioned again. Species 1 at time 2 (Fig. 6), would be considered a larva, and by saying that, one would mean that it is not yet a juvenile. Taxonomy is made to improve the self-consistency of our language. Deciding what a larva is, or what a juvenile is, are arbitrary conventions. Being so is not a failure: we expect them to be self-consistent and useful.

### Is this just a graph, or is this a phylogeny?

With the present method we are able to show for the first time the hierarchization power of parsimony mathematical procedures to organize through time the rise of organs for a set of species. However, the meaning has to be interpreted. Because descriptive embryology is related to evolution^31^, this graph is also a phylogeny: it is the finest graphical expression of ontophylogenesis^52,53^. The state of the art in theoretical biology fully authorizes such an interpretation. The two pillars of evolutionary theory—natural selection and descent with modification—are now included within the organismal body. Although the German embryologist Wilhelm Roux anticipated in 1881 that natural selection could explain the development and the functioning of the individual body^54,55^, one will have to wait more than a century to see the principle of natural selection among cells to come back within the soma to explain both stability of a metazoan and ageing^56^, as well as cancer^57–60^: « *cancer is a genetic disease fueled by somatic evolution* »^60^. Descent with modification explains paralogs in multigenic families within a same genome^61^, and partly explains cancer^62^. Descent with modification is obviously implied in papers reconstructing phylogeny of fixed somatic mutations within a single long-lived oak tree^63^, or phylogeny of cells within bodies of metazoans, e.g. within a developing zebrafish^64,65^, Xenopus^66^, or the ascidian *Ciona intestinalis*^67^. Natural selection and descent with modification being incorporated into the organismal development, the rise of the individual (ontogenesis) follows the same evolutionary processes as the rise of species (phylogenesis). We face a single global phenomenon, ontophylogenesis^52,53^ occurring at different scales, among cells of an individual and among individuals of a species (Table 3). As put by Moczek^31^, “*a theory of development should be nested within a theory of developmental evolution*”. In other words, in a fully nominalistic biology we would have already left the idealistic reification of the entities “individual” and “species”. Developmental stages are the taxonomic product of the segmentation of a process of change, just like species are. Then, in modern biology, it makes sense to interpret the hierarchical organization of ontogenetic time (shown here through the high C.I. and R.I. we obtained) into the framework of ontophylogenesis and to propose a phylogenetic segmentation of the ontogenetic time.

**Table 3:** During the twentieth century, there were two separate theories: one to understand the stabilization and change of the individual, and another one to understand the stabilization and change of the species. The former explained ontogenesis and the existence of individuals from a causative principle that was the genetic program. The latter explained phylogenesis and the existence of species from a causative principle that was descent with modification. Modern biology considers that only varying individual entities do exist, which undergo ontophylogenesis explained at all levels by natural selection and descent with modification.

Reified entity	Components	What is to be explained	What explains
Individuals	Cells	Ontogenesis	Genetic program
Species	Individuals	Phylogenesis	Descent with modification
None	Cells and individuals	Ontophylogenesis	Natural selection and descent with modification

## Methods

For this methodological proof-of-concept study, four teleostean freshwater species were chosen because an accurate description of their ontogenetic development was available and they display different reproductive characteristics^21,68^. We focused on the onset of a panel of organs, and organs presence at a particular time. Data for tench *Tinca tinca* were extracted from Peñáz^69,70^. Data for grayling *Thymallus thymallus* were extracted from Peñáz^71^. Data for huchen *Hucho hucho* were extracted from Peñáz & Prihoda^72^. Data from barbel *Barbus barbus* were extracted from Krupka^73^.

**Figure 7:**
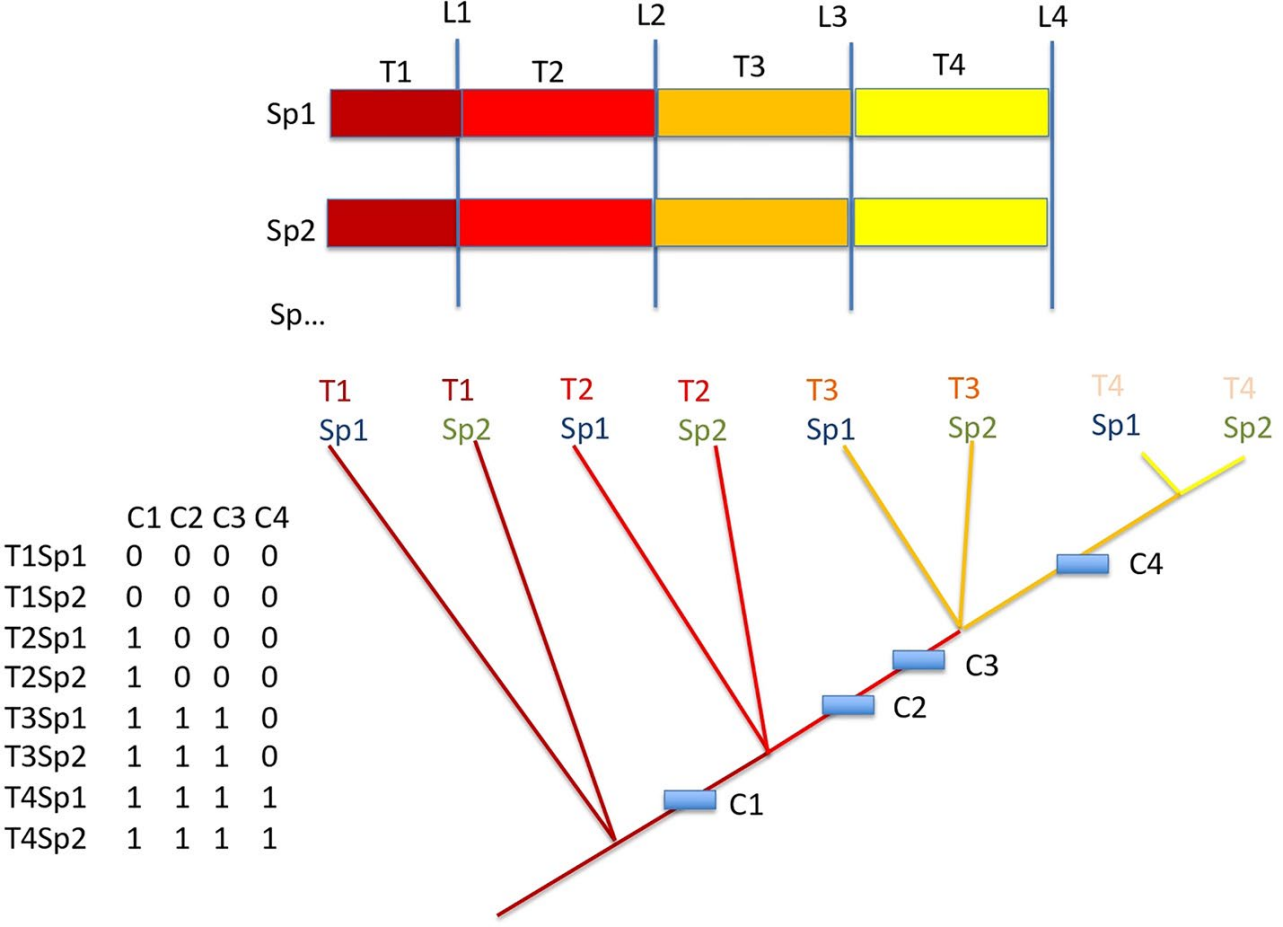
General methodological framework. Colored bars are developmental time for species 1 (Sp1) and species 2 (Sp2), and more species. L1, L2, etc. are arbitrary time landmarks measured as percentage of time (in degrees Celsius-days of development) of the full development from fecundation (0%) to the rise of lepidotrichia in pectoral fin rays (100%). Time segments T1, T2, etc. are defined between landmarks. The matrix at bottom-left records presence and absences of various organs and traits as characters (columns: C1, C2, etc.) for each OTU (line). An OTU is a given species at a given time segment. Bottom-right, the hierarchy of developmental time depicted with an oriented non cyclic connected graph (which is usually called a “tree”) obtained through a parsimony analysis of the matrix. This theoretical tree shows the same relative timing of the onset of organ for the two species.

Their developmental time frame was arbitrarily segmented in five parts, defined here as time landmarks: L1 = 0% of the developmental time, L2 = 25%, L3 = 50%, L4 = 75% and L5 = 100% (Fig. 7). As the developmental rate depends on temperature, and as we need to compare species, developmental time was normalized in degrees-Celsius-days. The 0% boundary is fecundation, the 100% boundary was arbitrarily chosen here as the onset of pectoral fin rays. Between time landmarks, we have four time segments T1 (from 0 to 25%), T2 (from 25 to 50%), T3 (from 50 to 75%) and T4 (from 75 to 100%). The time frame 25–50–75% was arbitrarily chosen. In order to compare more precisely the course of development across species, one could choose any another time frame. As an example, we have also chosen to explore more precisely the earliest moments of development with the time frames 5–10–50% and 5–10–20%.

Then we defined what we call “operational taxonomic units” (OTUs), which are a given species considered at a given time segment. T1Sp1 in Fig. 7 is the species no 1 considered at the time segment no 1, T2Sp2 is the species no 2 considered at the time segment no 2, T3Sp2 is the species no 2 considered at the time segment no 3, etc. For each OTU, we recorded in a data matrix the presence (coded 1) or the absence (coded 0) of a given organ, or structure, each of them being a column in the data matrix (Fig. 7), columns being classically called “characters” as in morphological data matrix^74,75^. The matrix is given in Table 1. The list of the 53 characters taken into account for this proof of concept is given in Table 2.

As most organs appear in a cumulative manner (e.g. the onset of skin pigmentation does not need to correspond to the loss of the heart, which itself does not need to correspond to the loss of the notochord), the nested hierarchy provided by a non-cyclical oriented connected graph is suitable for ordering organ onsets. Such graphs are usually called “rooted trees”. At each node of such trees, we need to identify what organs appear. Therefore, the matrix was then analyzed through a classical parsimony procedure, which maximizes the contiguity of branches having the same character states. Doing so, it finds the graph (or the tree) which minimizes the number of character changes, in other words which maximizes consistency of the whole set of characters through a single hierarchy^76–78^. This is what we need: as development is a cumulative process through time, this hierarchy is a time hierarchy^78^. Therefore, it is logical to define the outgroup (the root of the tree) at the boundary 0%: outgroups will be OTUs at 0% of their development (no traits). Potential loss of organs or traits during the development will appear as character reversals, which is classical in such analyses. The parsimony analysis was conducted using PAUP*^79^. The most parsimonious tree (in other words the most consistent hierarchy) was obtained through a branch-and-bound search (exact search^78^ on characters considered unweighted and unordered^74,75^. Character placement onto the tree was made under ACCTRAN optimization (favoring reversals when ambiguities in character optimization occurs).

**Figure 8:**
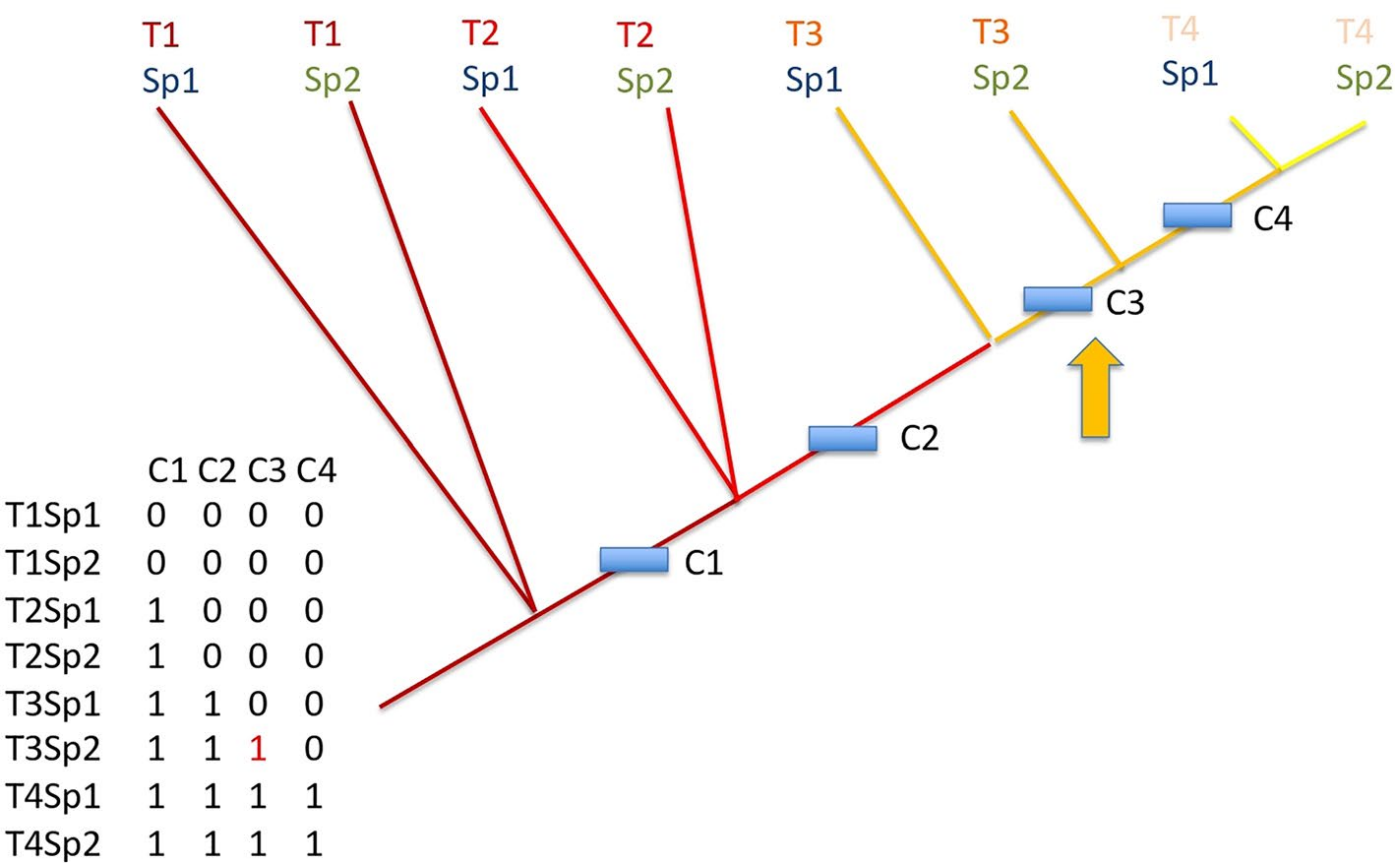
Same tree as Fig. 7, but with an heterochronic event. The relative timing of the onset of characters for the two species is not the same, as species 1 is late in gaining character 3 compared to species 2 which already has it at time segment 3.

If all developments among species follow the same timing of events (same relative timing in organ onsets, or “synchronic” development among species), we should theoretically obtain the tree shown in Fig. 7. Characters (i.e. traits or organs) are gained according to the same time hierarchy for all species. If a single heterochrony appears, for example species 1 is late at time 3 in gaining character 3 compared to all other species, one would obtain the tree shown in Fig. 8. Species 1 at time 3 does not have yet the character C3 that all other species (here just species 2) already have at that time.
